# Optimal range of gestational weight gain for singleton pregnant women: a cohort study based on Chinese specific body mass index categories

**DOI:** 10.1186/s12884-024-06592-y

**Published:** 2024-05-31

**Authors:** Yin Jia, Haili Jiang, Yuhui Fu, Yue Li, Huili Wang

**Affiliations:** 1 School of General Practice and Continuing Education, Capital Medical University, No. 10 You An Men Wai Xi Tou Tiao, Beijing, 100069 China; 2 Department of Obstetrics, Beijing Obstetrics and Gynecology Hospital, Beijing Maternal and Child Health Care Hospital, Capital Medical University, Beijing, 100026 China

**Keywords:** Gestational weight gain, Adverse pregnancy outcomes, Body mass index maternal health, Infant health

## Abstract

**Background:**

The purpose was to explore the optimal proportion of GWG in Chinese singleton pregnant women according to Chinese specific body mass index (BMI) categories.

**Methods:**

A retrospective cohort study with 16,977 singleton pregnant women was conducted. Among the including subjects, 2/3 of which were randomly imported into the training set for calculating the optimal GWG ranges using the percentile method, the Odd Ratio (OR) method, and the combined risk curve method. And another third of the subjects were used to evaluate the GWG ranges obtained. The detection rate of adverse outcomes of pregnant women was used to evaluate the applicability of GWG obtained. The range corresponding to the lowest detection rate is the recommended GWG range in this study.

**Results:**

According to the percentile method, the suitable GWG of pregnant women with underweight, normal weight, overweight or obesity before pregnancy were 12.0 ∼ 17.5 kg, 11.0 ∼ 17.0 kg, and 9.0 ∼ 15.5 kg, respectively. According to the OR method, the suitable GWG range were 11 ∼ 18 kg, 7 ∼ 11 kg, and 6 ∼ 8 kg, respectively. According to the combined risk curve method, the suitable GWG range were 11.2 ∼ 17.2 kg, 3.6 ∼ 11.5 kg, and − 5.2 ∼ 7.0 kg, respectively. When the GWG for different BMI categories were 11 ∼ 18 kg, 7 ∼ 11 kg, and 6 ∼ 8 kg, the pregnant women have the lowest detection rate of adverse maternal and infant outcomes.

**Conclusions:**

The recommended GWG based on this study for underweight, normal, overweight or obese pregnant women were 11 ∼ 18 kg, 7 ∼ 11 kg, and 6 ∼ 8 kg, respectively.

**Supplementary Information:**

The online version contains supplementary material available at 10.1186/s12884-024-06592-y.

## Background

The metabolic and nutritional status of pregnant women during pregnancy were reported to increase the risk of cardiovascular related diseases or other chronic diseases in adulthood in many laboratory and epidemiological studies [1–5]. Pregnancy, as a controllable link affecting pregnancy outcome, has been reported to be essential for the short-term and long-term health of mothers and infants [6, 7]. The pregestational body mass index (BMI) and gestational weight gain (GWG) were proved to be associated with the placental development, blood sugar control, glucose tolerance, and insulin resistance [8–11]. Multiple cytokines are involved in the process of imbalanced gestational diabetes mellitus (GDM) insulin resistance and the placental development [10–15], affecting pregnancy outcomes. Researches has shown that pregnant women who experience inappropriate weight gain during pregnancy have significantly reduced serum Nrf2 and PLGF levels, and increased expression of PTH-rP in the placental issue, which impairs placental development and increases the risk of adverse pregnancy outcomes such as GDM, preeclampsia, and so on [11–15].

GWG ranges of mothers-to-be was believed to be an important predictor of pregnancy outcomes [16, 17]. Inappropriate GWG are of high risk with suffering GDM, hypertensive disorder complicating pregnancy (HDP), and other adverse pregnancy outcomes [18–21]. Accordingly, it is very necessary to explore the suitable GWG ranges to obtain good pregnant outcomes, and the exploration of the appropriate range of weight gain during pregnancy has been ongoing [22–27].

In the 1990s, Institute of Medicine (IOM) proposed that weight management during pregnancy should be basic on the level of women’s pre-pregnancy BMI, and put forward a guideline for women’s weight gain during pregnancy in the United States [28]. The guideline was updated in 2009 and has become the most widely used guideline on the scope of weight gain during pregnancy in the world [29], which was used as a guidance for preconception and prenatal care in China (2018) [30]. However, in view of the differences in race, environment, economic development level, medical service level, living habits, etc., the applicability of IOM 2009 GWG guidelines to pregnancy weight gain in other countries is controversial [7, 31–33]. This guideline has been demonstrated unsuitable for all Chinese women [34]. Many researches in different countries continued to explore appropriate gestational weight gain based on national data on pregnant women [31–33, 35, 36], and the statistical methods used and the GWG range recommended were also different.

Maternal and Child Health Standards Professional Committee of National Health Commission (NHC) have issued the Standard of Recommendation for Weight Gain during Pregnancy Period for Chinese women in 2022 [36], which is the first authoritative GWG guidelines specifically for Chinese pregnant women. This NHC GWG guideline provides the range of weight gain for pregnant women in the early and overall stages of pregnancy, as well as the rate of weight gain in the middle and late stages of pregnancy, while no specific research method and process. Considering that the IOM guidelines are constantly being revised with the development of society, it is necessary to continuously improve and optimize most guidelines.

Therefore, the purpose of this retrospective study is to create the optimal GWG range for Chinese singleton pregnant women based on Chinese-specific BMI classification [26] using three different methods, so as to provide the foundation and evidence for further improvement of the NHC GWG guidelines.

## Methods

### Study populations

**Fig. 1:**
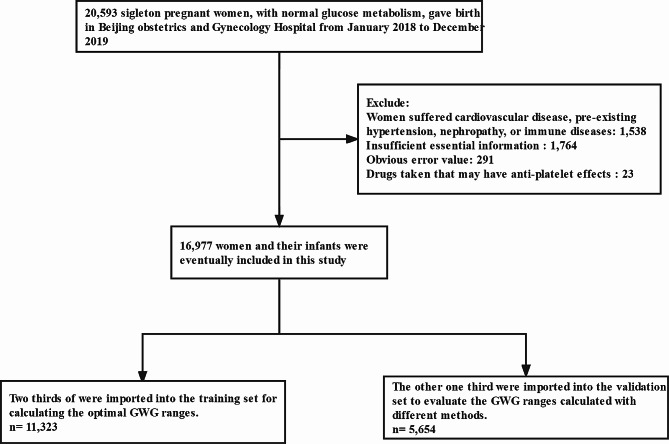
Flowchart for inclusion and exclusion of study populations

The research subjects were singleton pregnant women who underwent labor in Beijing Obstetrics and Gynecology Hospital from January 2018 to December 2019. Beijing Obstetrics and Gynecology Hospital is the largest obstetrics and gynecology hospital in North China, accounting for about one-tenth of all newborns in Beijing each year. Inclusion criteria: (1) Pregnant women who had their maternity check-ups and underwent labor at Beijing Obstetrics and Gynecology Hospital; (2) Healthy pregnant women with no history of cardiovascular disease, hypertension, diabetes, or hematologic diseases; (3) Pregnant women with no clear family history of diabetes or hypertension; (4) Singleton pregnant women with normal glucose metabolism. Exclusion criteria: (1) Lethal fetal malformation cases; (2) Stillbirth cases; (3) Pregnant women diagnosed with gestational diabetes mellitus; (4) Cases with missing information of maternal height, maternal weight, gestational age, apgar score, birth weight, or birth length. The process of inclusion and exclusion of research subjects is detailed in Fig. 1. This study was reviewed and approved by the Ethics Committee of Beijing Obstetrics and Gynecology Hospital, and the approval number is 2022-KY-019-01.

### Study design and grouping

A column of random numbers was generated in SPSS using the random number seed “1234567890” and arranged in ascending order. Accordingly, the 1st to 11323rd cases were imported into the training set for calculating the optimal GWG ranges using three methods. The 11324th to 16977th cases (5,654 cases in total) were imported into the validation set to evaluate the GWG ranges calculated with different methods. The pre-pregnancy BMI of pregnant women was classified according to the recommended BMI criteria for Chinese adults proposed by the Working Group on Obesity in China (WGOC) [37], that is, BMI < 18.5 kg/m^2^ is for underweight, 18.5 kg/m^2^ < BMI < 24 kg/m^2^ is for normal weight, BMI > 24 kg/m^2^ is for overweight or obesity.

### Anthropometric measurement

The relevant data required for this study were obtained through the electronic medical record system of Beijing Obstetrics and Gynecology Hospital. Baseline data included: age of pregnant women, weight before pregnancy, height, gestational weeks of delivery, parity, mode of delivery, birth weight and gender of newborns. Pregnancy complications and adverse pregnancy outcomes include: hypertensive disorder complicating pregnancy, preeclampsia, cesarean section, low birth weight (LBW), macrosomia, small for gestational age (SGA), large for gestational age (LGA), premature delivery, postpartum hemorrhage and neonatal asphyxia.

Pre-pregnancy BMI (kg/m^2^) was calculated as [pre-pregnancy weight (kg)]/ [height^2^ (m^2^)]. Weight gain during pregnancy (kg) was calculated as weight before delivery (kg) minus weight before pregnancy (kg). The gestational weeks of delivery were calculated by the competent doctor according to the time interval from the first day of the last menstrual cycle of the pregnant woman to the time of delivery, and recorded in “weeks”. The weight before delivery was measured by the midwife before the parturient entered the delivery room and recorded in “kg”. The weight of the newborn was weighed by the midwife in the delivery room with the baby scale within 1 h after the birth of the newborn and recorded in “g”. The length of the newborn was measured by the midwife in the delivery room with a soft ruler within 1 h after the birth of the newborn and recorded in “cm”.

### Definition of outcomes

Main outcomes included cesarean section, HDP, premature birth, LBW infants, macrosomia, SGA, and LGA. If the research subject experienced one or more events in the main outcomes, it can be considered that the composite endpoint has occurred. Hypertensive disorder complicating pregnancy is hypertension diagnosed before pregnancy or newly diagnosed hypertension during gestation [38]. Preterm births are defined as the ones at less than 37 weeks of gestation [39]. LBW refers to live-born neonates with a birth weight below 2,500 g [40]. Macrosomia refers to live-born neonates with a birth weight above 4,000 g [41]. SGA refers to neonates with a birth weight below the 10th percentile of the average weight of infants of the same sex and gestational age [42]. LGA refers to neonates with birth weight above the 90th percentile of the average weight of infants of the same sex and gestational age [42].

Other outcomes included preeclampsia, postpartum hemorrhage. Preeclampsia is characterized by hypertension and proteinuria in the second half of pregnancy, which subsides soon after delivery [43]. Postpartum hemorrhage is defined as the blood loss during vaginal delivery is more than 500 ml, and more than 1000 ml during cesarean section, within 24 h after delivery of the fetus [44].

### Statistical analysis

#### Analytical method

Three analytical methods were used to investigate the appropriate gestational weight gain. The percentile method in this study takes the population with good maternal and infant outcomes in the training set as the reference population, and refers to the research methods reported in the previous literature [31, 45]. According to the 25th to 75th percentile of the weight gain of pregnant women with good maternal and infant outcomes in the database, the suitable weight gain range during pregnancy is taken. Good maternal and infant outcomes were defined as: no pregnancy induced hypertension, cesarean section with medical indications, small for gestational age infants, large for gestational age infants, low birth weight infants, macrosomia, or premature birth.

The percentile method is the most common method for calculating the range of medical reference value, but it does not include data on pregnant women with adverse pregnancy outcomes. The odd ratio (OR) method and combined risk curve method calculate the appropriate GWG by minimizing the joint risk of composite endpoint events, including more adverse pregnancy outcome events.

The OR method refers to the method used by professor Cedergren et al. to analyze the suitable weight gain during pregnancy in Sweden in 2007 [16]. Pregnant women in different BMI groups were grouped into different weight gain classes every 1 kg weight gain (the corresponding weight gain class of 10 kg group is 10 kg ≤ x<11 kg) and those with a small number of people in some weight gain intervals are grouped into one group, according to the methods used in previous studies to analyze the appropriate GWG [16]. A multivariate logistic regression analysis was conducted to calculate the odds ratios (ORs) and 95% confidence intervals (CIs) of adverse pregnant outcomes, after adjusting for maternal age, prepregnancy BMIs, gestational weeks of delivery and parity. The establishment of optimal GWG range was based on weight gain intervals corresponding to an OR values less than 1 and the upper and lower cutoff value of 95% CIs.

The combined risk curve method is the method used to analyze the appropriate weight gain during pregnancy with reference to previous studies [32, 33], and has been optimized in combination with the clinical actual situation of Chinese pregnant women. Exponential function model was used to improve the fitness between GWG and the predicted probability of a single adverse outcome. And quadratic function model was used to improve the fitness between GWG and the total predicted probability. The range of weight gain during pregnancy recommended by this method is the range corresponding to the lowest combined risk increase of no more than 1%.

#### Statistical description and analysis

Epidata 3.0 was used to record the data, and SPSS 26.0 was used for statistical analysis. T-test was used for continuity variables, and χ^2^-test was used for classification variables. The baseline characteristics and the incidence of adverse outcomes of pregnant women in the training set and the validation set were compared. After the normality test of the continuous variables, measures such as those conforming to a normal distribution were statistically described as mean ± standard deviation (mean ± SD), and those not conforming to a normal distribution were described as median and interquartile range. The count data were statistically described as frequency (n) and percentages (%). The continuous variables were analyzed with ANOVA, and categorical variables were analyzed with the chi-square test. Differences were considered statistically significant at *P* < 0.05.

Using the data of pregnant women in the training set, three analytical methods were used to calculate the suitable weight gain range during pregnancy. Using the data of pregnant women in the validation set, the McNemar’s paired chi-square test was used to evaluate the above three ranges with “weight (kg)” as the index. The detection rates of each adverse outcome and composite endpoint event within the recommended reference range were calculated and compared respectively. The detection rates of adverse maternal and infant outcomes of pregnant women with appropriate weight gain obtained by different methods were compared. The range corresponding to the lowest detection rate is the recommended GWG range in this study.

## Results

Of all the women giving birth during the study period, 16,977 pregnant women were eventually included in this study.

### Basic characteristics of the research subjects

The mean age of included mothers was 32.0 ± 3.8 years old and the mean GWG was 14.3 ± 4.9 kg. The rate of nulliparity was 12,172 (71.7%). Among the 16,977 newborns, 8771 (51.7%) were boys and 8206 (48.3%) were girls. The mean birth weight of included infants was 3343.3 ± 480.4 g and the mean birth height was 50.0 ± 2.0 cm. According to the BMI classification standard recommend by WGOC [21], 2,298 (13.5%) of the subjects were underweight before pregnancy, 11,379 (67.0%) were in normal weight before pregnancy, 3,300 (19.4%) were overweight or obesity before pregnancy. The baseline characteristics of the pregnant women included in this study are summarized in Table 1.

**Table 1:** Basic characteristics of the included mother-infant pairs (*n* = 16,977)

Variables	$\bar x \pm s$ /*n*(%)
Mother’s age (years), mean ± SD	32.0 ± 3.8
Mother’s height (cm), mean ± SD	163.0 ± 5.0
Pre-pregnancy weight (kg), mean ± SD	57.5 ± 8.9
Mother’s pre-pregnancy BMI (kg/m^2^), mean ± SD	21.64 ± 3.13
Gestational weight gain (kg), mean ± SD	14.3 ± 4.9
Gestational week (weeks), mean ± SD	38.9 ± 1.7
Pre-pregnancy BMI classification n(%)	
Underweight (< 18.5 kg/m^2^)	2298 (13.5%)
Normal weight (18.5–23.9 kg/m^2^)	11,379 (67.0%)
Overweight (25.0–27.9 kg/m^2^)	2578(15.2%)
Obesity (≥ 28.0 kg/m^2^)	722(4.3%)
Parity n(%)	
Primiparity	12,172 (71.7%)
Multiparity	4805 (28.3%)
Gender of newborn n(%)	
Girl	8206 (48.3%)
Boy	8771 (51.7%)
Neonatal length (cm), mean ± SD	50.0 ± 2.0
Neonatal weight (g), mean ± SD	3343.3 ± 480.4

This study adopted the research design of training set and validation set. Pregnant women were randomly divided into two sets, of which 66.7% (11,323 cases) were included in the training set, and another 33.3% (5654 cases) were included in the validation set. The baseline characteristics of the two sets showed no obvious difference, and the incidence of adverse maternal and infant outcomes in the two sets showed no statistically significant difference (shown in Table 2).

**Table 2:** Comparison of basic characteristics and outcomes between two data set

Variables	Training set (*n* = 11,323)	Validation set (*n* = 5654)	t/χ^2^	*P*
Mother’s age (years), mean ± SD	32.0 ± 3.9	32.1 ± 3.8	-0.369	0.712
Mother’s height (cm), mean ± SD	163.0 ± 5.0	163.0 ± 5.0	0.012	0.991
Pre-pregnancy BMI (kg/m^2^), mean ± SD	21.7 ± 3.2	21.6 ± 3.1	1.504	0.133
Gestational weight gain (kg), mean ± SD	14.3 ± 4.9	14.3 ± 4.9	-0.067	0.946
Gestational week (weeks), mean ± SD	38.9 ± 1.7	38.9 ± 1.7	-0.578	0.563
Neonatal weight (g), mean ± SD	3340.2 ± 480.4	3349.4 ± 480.4	-1.171	0.471
Neonatal length (cm), mean ± SD	50.0 ± 2.0	50.0 ± 2.0	-0.727	0.977
Primiparity n(%)	8142 (71.9%)	4030 (71.3%)	0.737	0.391
Cesarean delivery n(%)	3392 (30.0%)	1761 (31.1%)	2.523	0.112
CS with medical indications n(%)	2379 (21.1%)	120 (21.3%)	0.231	0.631
Preeclampsia n(%)	545 (4.8%)	262 (4.6%)	0.268	0.605
Postpartum hemorrhage n(%)	663 (5.9%)	314 (5.6%)	0.633	0.426
Gender of newborn (boy) n(%)	5855 (51.7%)	2916 (51.6%)	0.027	0.868
Preterm birth n(%)	552 (4.9%)	260 (4.6%)	0.633	0.426
Low birth weight n(%)	402 (3.6%)	198 (3.5%)	0.026	0.872
Macrosomia n(%)	728 (6.4%)	393 (7.0%)	1.663	0.197
Small for gestational age n(%)	581 (5.1%)	305 (5.4%)	0.528	0.467
Large for gestational age n(%)	1831 (16.2%)	954 (16.9%)	1.357	0.244
Composite endpoints n(%)	4959(43.8%)	2510(44.4%)	0.546	0.460

BMI: body mass index

### The optimal range of gestational weight gain

#### Percentile method

Among the 11,323 pregnant women in the calculation database, 6364 had good maternal and infant outcomes. According to the 25th to 75th percentile distribution of pregnancy weight gain level under different pre pregnancy weight classification, the recommended ranges of pregnancy weight gain for pregnant women with low pre pregnancy weight, normal weight, overweight or obesity were 12.0 ∼ 17.5 kg, 11.0 ∼ 17.0 kg, 9.0 ∼ 15.5 kg respectively (shown in Table 3).

**Table 3:** Percentile distribution of GWG of pregnant women in training set

Pre-pregnancy BMI classification	P2.5	P5	P10	P25	P50	P75	P90	P95	P97.5
Underweight	7.0	8.5	10.0	12.0	15.0	17.5	20.0	22.0	24.0
Normal weight	6.0	7.2	9.0	11.0	14.0	17.0	20.0	21.5	23.0
Overweight or obesity	2.0	4.5	6.0	9.0	12.0	15.5	19.0	21.0	24.0

#### OR method

**Fig. 2:**
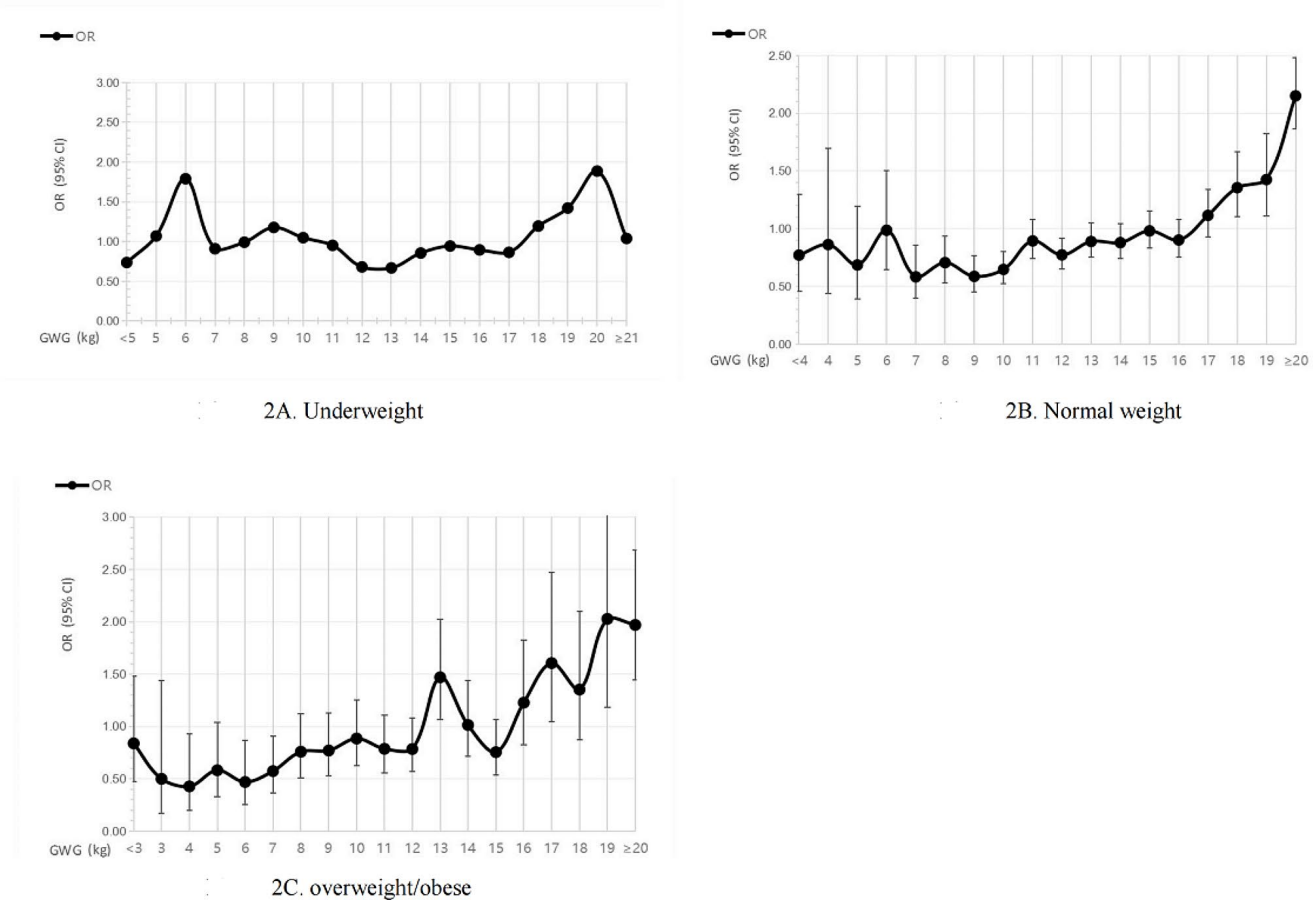
OR value of composite endpoint under with increasing gestational weight gain under Chinese specific BMI classifications. Underweight (**A**), normal weight (**B**), overweight or obesity women (**C**)

For pregnant women of underweight bef percentile method e pregnancy, the weight gain classes with OR less than 1 were 11.0 ∼ 18.0 kg. Therefore, the optimal GWG range for underweight women were 11.0 ∼ 18.0 kg (shown in Fig. 2-A). The optimal GWG range for normal weight, and overweight or obesity women was 7.0~11.0 kg (shown in Fig. 2-B) and 6.0~8.0 kg (shown in Fig. 2-C), respectively, whose upper and lower cutoff levels of the 95%CIs were below 1 for each maternal weight gain classes.

#### Combined risk curve method

**Fig. 3:**
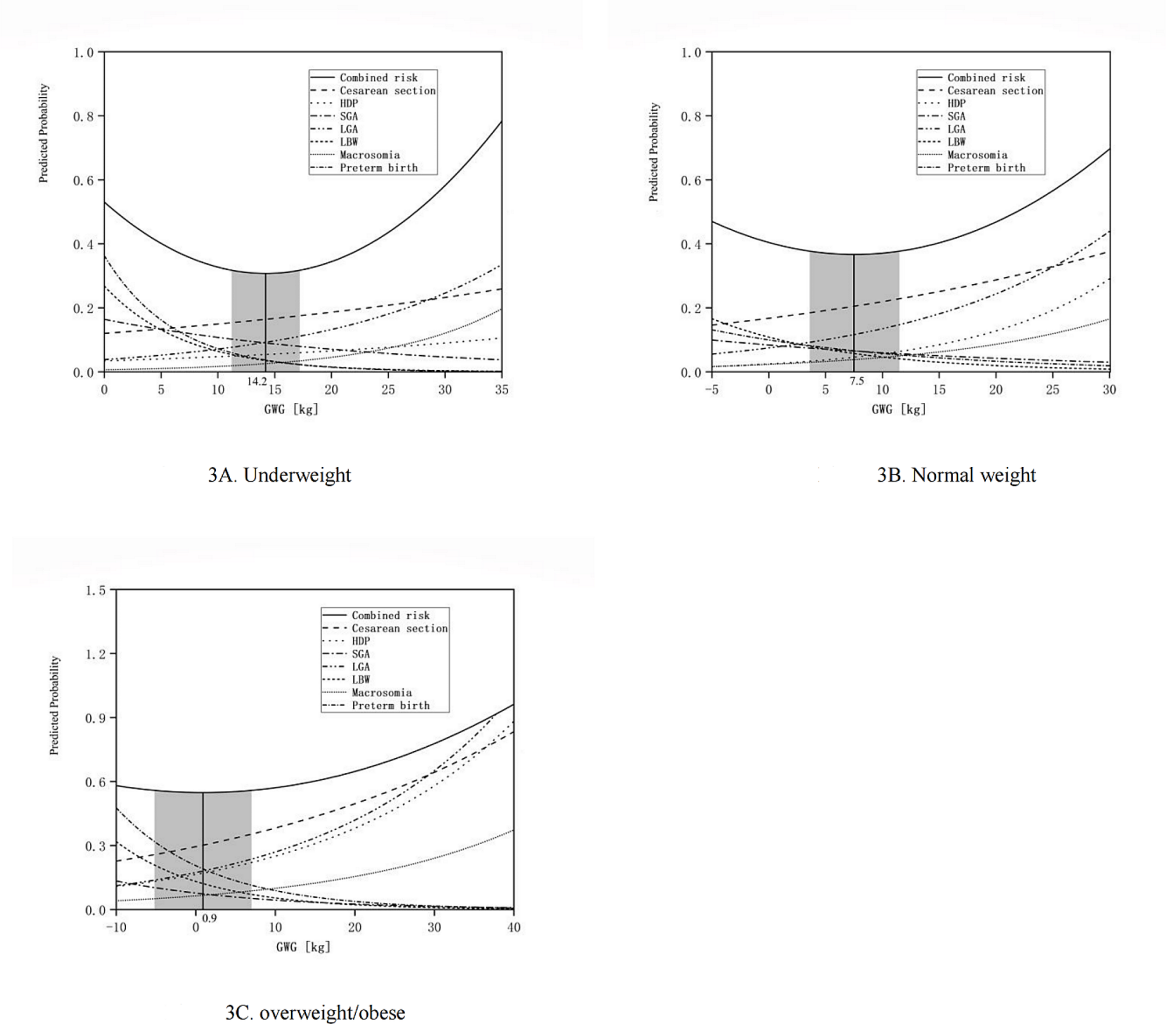
Predicted probabilities of cesarean section, hypertensive disorder complicating pregnancy, small for gestational age, large for gestational age, macrosomia, low birth weight, preterm birth, with increasing gestational weight gain, stratified by Chinese specific BMI categories. Underweight (**A**), normal weight (**B**), overweight or obesity women (**C**)

For underweight women before pregnancy, the lowest predicted probability of composite adverse pregnant outcomes corresponded to the weight of 14.2 kg, the recommended range were 11.2~17.2 kg (Fig. 3-A). For normal weight women before pregnancy, the lowest total predicted probability of composite adverse pregnant outcomes corresponded to the weight of 7.5 kg, the recommended range were 3.6~11.5 kg (Fig. 3-B). For overweight or obesity women before pregnancy, the lowest total predicted probability of the combined risk of adverse outcomes corresponded to the weight of 0.9 kg, the recommended range were − 5.2~7.0 kg (Fig. 3-C).

### Evaluation of the gestational weight gain ranges

Single adverse maternal and infant outcomes and composite endpoint events were the lowest in pregnant women with appropriate weight gain obtained by the OR method. Single adverse maternal and infant outcomes and composite endpoint events were the highest in pregnant women with appropriate weight gain obtained by the percentile method. The detection rate of adverse maternal and infant outcomes under different methods was shown in Table 4. Therefore, the recommended optimal GWG ranges for underweight, normal, and overweight/obese pregnant women were 11.0 to 18.0 kg, 7.0 to 11.0 kg, and 6.0 to 8.0 kg, respectively, according to the OR method.

**Table 4:** Comparison of detection rates of adverse maternal and infant outcomes

Maternal and neonatal outcomes	OR method	Percentile method	Combined risk curve method
HDP (*n* = 584)	71 (12.1%)	269 (46.0%)	109 (18.6%)
Preeclampsia (*n* = 262)	34 (12.9%)	114 (43.5%)	45 (17.1%)
CS with medical indications (*n* = 1206)	180 (14.9%)	656 (54.3%)	265 (21.9%)
Postpartum hemorrhage (*n* = 314)	53 (16.8%)	164 (52.2%)	82 (26.1%)
Preterm birth (*n* = 260)	63 (24.2%)	131 (50.3%)	98 (37.6%)
Low birth weight (*n* = 198)	50 (25.2%)	90 (45.4%)	89 (44.9%)
Macrosomia (*n* = 393)	32 (8.1%)	196 (49.8%)	53 (13.4%)
Small for gestational age (*n* = 305)	78 (25.5%)	159 (52.1%)	108 (35.4%)
Large for gestational age (*n* = 954)	112 (11.7%)	486 (50.9%)	172 (18.0%)
Neonatal asphyxia (*n* = 68)	11 (16.1%)	33 (48.5%)	23 (33.8%)
Composite endpoints (*n* = 2510)	401 (15.9%)	1317 (52.4%)	591 (23.5%)

HDP: hypertensive disorder complicating pregnancy; CS: cesarean section

## Discussion

Three different analysis methods were adopted to explore the optimal weight gain ranges based on Chinese specific pre-pregnancy BMI categories in this large, population-based cohort study. The recommended optimal GWG ranges for underweight, normal, and overweight/obese pregnant women were 11.0 to 18.0 kg, 7.0 to 11.0 kg, and 6.0 to 8.0 kg, respectively, after evaluating the ranges obtained by those three methods.

The GWG recommendations varied by national origin and intrinsic ethnic. Taking pregnant women with normal weight before pregnancy for example, the recommended range of weight gain in Sweden in 2007 was 2 ∼ 10 kg [16], and that in Germany was 2 ∼ 18 kg [32]. The recommended range of Singapore in 2014 was 7.7 ∼ 18.8 kg [24]. In 2017, the weight gain weight range recommended by South Korea was 11.5 ∼ 21.5 kg [33], and that recommended by Japan was 9.7 ∼ 10.4 kg [46]. The optimal GWG ranges recommended in this study were lower and narrower than that recommended by Sae Kyung Choi for the Korean population, based on the joint risk curve method [33]. The recommended GWG ranges in this study were a little higher than that recommended by Cedergren for the Swedish population, according to the OR method [16]. This may be related to differences in pre-pregnancy BMI classification, outcome events, religion, living environment, economic status, and diet structure, and geographical location between pregnant women in China and other countries.

In terms of the optimal GWG ranges, our findings are not consistent with the results from previous studies on Chinese pregnant women [7, 47]. The differences may be attributed to the different outcomes included in the studies. Both all-cause cesarean section and gestational diabetes were included in the two previous studies [7, 20], while not included in this study. Firstly, the cesarean section rate of pregnant women in China has been rising in the past 10 years, reaching 36.7% by 2018, ranking first in Asian countries [48], far higher than the 15% reference recommended by World Health Organization (WHO) [49]. The high cesarean section rate in China is mainly related to the fact that some pregnant women are afraid of pain, scarring or other social and psychological factors, and also related to the lagging development of midwifery in some areas. Therefore, taking all-cause cesarean section into outcome indicators may cause some bias to the results of the appropriate GWG range. Secondly, previous two studies [7, 47] showed that weight gain during pregnancy is a protective factor for the incidence of gestational diabetes and pregnant women with insufficient GWG are more likely to develop gestational diabetes, which is contrary to the physiological mechanism of pregnancy [50]. It can be supposed that the diagnosis time of gestational diabetes is generally 24–28 weeks of pregnancy, in which the pregnant women diagnosed with gestational diabetes would reduce the intake of carbohydrate and fat in daily diet and increase exercise in daily life, so that the total GWG would lower than those diagnosed without gestational diabetesand [51–53]. In this case, taking gestational diabetes as one of the outcomes may reduce the preciseness of the selection of outcome indicators. In addition to the different outcome indicators, there are many other factors may influence the range for GWG in various studies.

Different statistical methods used in different studies may also cause differences in results. Even studies using the same statistical method recommend a completely different range of body mass, which may be related to the different inclusion outcomes. In terms of the outcome of the study, outcomes related to neonatal weight, such as SGA, LGA, low birth weight infants and macrosomia, are the most commonly used outcome indicators to calculate the range of weight gain during pregnancy, and pregnancy induced hypertension or preeclampsia are the most commonly used outcome indicators to minimize its risk [7, 21, 32, 34–36].

BMI classification criteria used in different studies may also related to different results. The distribution of pre-pregnancy BMI categories for pregnant women in this study was different with other studies to some extent [7, 54]. Some studies use Chinese specific BMI categories by WGOC [2, 7, 47], some studies use the BMI categories recommended by WHO [55–57], and some studies use their own pre pregnancy BMI classification to study the appropriate range of weight gain [58–60]. Therefore, different BMI classification standards for prepregnancy women may have a certain impact on the results.

The sample size of this study was large, and the samples were all from Beijing Obstetrics and Gynecology Hospital, with the highest number of deliveries and high-risk pregnancies in Beijing, which greatly increased the sample diversity. In addition to the calculation database, a verification database was designed to validate the recommended GWG ranges, which greatly increased the scientific validity of the research methodology and the credibility of the results. The detection rate of all the adverse pregnant outcomes based on the GWG recommendations proposed in this study was lower than those based on the NHC GWG guidelines (shown in Appendix 1), which indicates that the range of the NHC guidelines could be optimized in depth. This current study provided theoretical basis and evidence for the optimization of the guideline to some extent.

However, this study has inevitable limitations. As a single-center retrospective study, the sample representation may have some bias. In addition, due to the small proportion of pregnant women obese before pregnancy, the optimal GWG ranges for them require further calculation and verification with an expanded sample size. Future prospective multi-center studies were encouraged to combined with clinical experience and different statistical research methods to explore the appropriate range of weight gain during pregnancy more in depth.

## Conclusion

The optimal GWG range recommended by this study was 11~18 kg for underweight pregnant women, 7~11 kg for normal weight pregnant women, and 6~8 kg for overweight or obesity pregnant women. The GWG recommendation in this study can help reduce the risk of adverse pregnancy outcomes to a certain extent and provide references for clinicians to manage the weight of pregnant women. Weight gain during pregnancy should be scientifically controlled within the appropriate ranges to reduce the incidence of adverse pregnancy outcomes and promote maternal and infant health.

### Electronic supplementary material

Below is the link to the electronic supplementary material.

Supplementary Material 1

## Data Availability

The datasets generated and/or analysed during the current study are not publicly available due to the containing information that could compromise the privacy of research participants but are available from the corresponding author on reasonable request.
