# Supplementary material for: Optimal range of gestational weight gain for singleton pregnant women: a cohort study based on Chinese specific body mass index categories
Source: BMC Pregnancy Childbirth. 2024 May 31;24:399. doi: 10.1186/s12884-024-06592-y (PMC11143601; doi:10.1186/s12884-024-06592-y)
Supplement: Supplementary file 1 — Supplementary Material 1 [file 12884_2024_6592_MOESM1_ESM.docx]

Appendix 1. Comparison between our proposed GWG ranges and NHC guidelines

| Maternal and neonatal outcomes | Our proposed GWG ranges | NHC GWG |
| --- | --- | --- |
| HDP (n=584) | 71 (12.1%) | 167（28.5%） |
| Preeclampsia (n=262) | 34 (12.9%) | 78（29.7%） |
| CS with medical indications (n=1206) | 180 (14.9%) | 445（36.8%） |
| Postpartum hemorrhage (n=314) | 53 (16.8%) | 125（39.8%） |
| Preterm birth (n=260) | 63 (24.2%) | 123（47.3%） |
| Low birth weight (n=198) | 50 (25.2%) | 99（50.0%） |
| Macrosomia (n=393) | 32 (8.1%) | 105（26.7%） |
| Small for gestational age (n=305) | 78 (25.5%) | 145（47.5%） |
| Large for gestational age (n=954) | 112 (11.7%) | 304（31.8%） |
| Neonatal asphyxia (n=68) | 11 (16.1%) | 25（36.7%） |
| Composite endpoints (n=2510) | 401 (15.9%) | 925（36.8%） |

HDP, hypertensive disorder complicating pregnancy; CS, cesarean section.
